# The Potential Risk Factors Relevant to Lateral Epicondylitis by Wrist Coupling Posture

**DOI:** 10.1371/journal.pone.0155379

**Published:** 2016-05-12

**Authors:** Su-Ya Lee, Hsiao-Feng Chieh, Chien-Ju Lin, I-Ming Jou, Li-Chieh Kuo, Fong-Chin Su

**Affiliations:** 1 Department of Biomedical Engineering, National Cheng Kung University, Tainan, Taiwan; 2 Department of Orthopedics, National Cheng Kung University Hospital, Tainan, Taiwan; 3 Medical Device Innovation Center, National Cheng Kung University, Tainan, Taiwan; 4 Department of Occupational Therapy, National Cheng Kung University, Tainan, Taiwan; Mayo Clinic Minnesota, UNITED STATES

## Abstract

The use of awkward wrist postures and unskilled techniques might induce lateral epicondylitis. This study thus investigated the effects of wrist deviation combined with extension and movement velocity on the dynamic performances of the wrist muscles during the coupling posture via a custom-made bi-planar isokinetic dynamometer. Thirty subjects were recruited to perform the isokinetic testing. We measured the muscle strengths and activities for the wrist extensors and flexors during concentric and eccentric contractions at three movement velocities, 30°s^-1^, 90°s^-1^, and 180°s^-1^, combined with three wrist postures, neutral position (NP), radial deviation (RD), and ulnar deviation (UD). The root mean square (RMS) of the electromyographic signal in the extensor digitorum communis (EDC), normalized peak torque of extensors, and ratio of normalized peak torque between wrist extensors and flexors, were all greater in the NP than RD and UD in both contractions. The ratio of RMS between EDC and flexor digitorum superficialis (FDS) had a significantly greater value in RD than UD during the concentric contraction. The EDC showed significantly higher activity at the fast velocity in both contractions. Nevertheless, a significantly higher RMS of the electromyographic signal between EDC and FDS and the ratio of strength between wrist extensors and flexors were found at slow velocity in both contractions. The wrist deviation combined with extension and movement velocity of the wrist joint should thus be considered as influential factors which might alter the dynamic performances, and may result in further injury of the elbow joint.

## Introduction

Lateral epicondylitis results from various factors, such as repetitive stressful tasks, overuse and poor posture [1, 2], and usually causes chronic pain over the upper limbs as well as functional disabilities. According to previous studies, most patients with lateral epicondylitis were among the population of manual workers and athletes, who often make repetitive movements of the forearm muscles against resistance [1–3]. The grip strength [4], balance of muscle activity in the agonist and antagonist [5, 6], and muscle strength of the upper extremities [5–7] are common evaluations used to measure lateral epicondylitis. The measurement of isokinetic contraction can provide information about muscular performance in a specific movement velocity and adaptations of muscular strength in sports activities, working movements, or rehabilitation interventions. The assessment of isokinetic strength has also been widely used to investigate the changes in strength in patients with lateral epicondylitis [8], but there are few studies presenting information on such dynamic performances based on isokinetic testing with eccentric and concentric contractions at different movement velocities and coupling postures in the wrist joint. The data on dynamic performances that is obtained from isokinetic testing, such as muscle strength and activity at the wrist joint with different coupling postures, can provide information to assess the severity of lateral epicondylitis, as well as improve rehabilitation interventions.

The coupling posture of the wrist joint plays an important role in the skilled performance of the hand during in many daily living, sports and workplace activities. Garge et al. showed that dart throwing, hammering, basketball shooting, and pouring all include coupled wrist motions, and the kinematic path is from extension-radial deviation to flexion-ulnar deviation [9]. So far, however, no studies have investigated the strength and muscle activity of wrist extensors and flexors at the coupling posture during isokinetic contraction, because commercial dynamometer mechanisms can only set the joint position in a single plane of motion. Nevertheless, novice tennis players have more ulnar deviation/flexion during backhand strokes and generate a longer time of eccentric contraction than professional players [10], and these awkward postures and unskilled techniques might induce lateral epicondylitis. Based on isokinetic performance at the coupling movement, clinicians or trainers can provide different treatment protocols after making a quantified assessment of functional performance in healthy subjects and patients with upper extremity disorders.

The aims of this study were as follows: (1) to investigate the effects of wrist deviation combined with extension and movement velocity on different muscle contraction types by measuring the strength and muscle activity of the wrist joint at the coupling posture via a custom-made bi-planar isokinetic dynamometer (BID); and (2) to obtain information on the wrist extensor-flexor ratio in strength and muscle activity at different wrist deviations combined with extension and movement velocities. The knowledge obtained in this study may provide evidence-based data to better understand wrist muscle recruitment patterns during the wrist coupling posture by evaluating healthy subjects, since this can provide normative information to assess the functioning of patients with lateral epicondylitis. By investigating the effects of wrist coupling posture and movement velocity, it is possible to better understand the related changes in muscle strength and activity, which could then be used to find the risk factors for lateral epicondylitis. The results of this study may lead to the development of better interventions for patients with lateral epicondylitis, thus addressing the problem of muscle imbalance by establishing data on the wrist extensor-flexor ratio in strength and muscle activity.

## Materials and Methods

### Participants

Thirty healthy subjects (14 males and 16 females; age: 23.74 ± 2.16 years, body mass: 63.13 ± 11.79 kg, height: 167.68 ± 9.68 cm) were recruited to perform the isokinetic testing. All subjects were right hand dominant. The inclusion criteria for this study were as follows: no present discomfort or pain in the forearm and wrist, no history of upper extremity musculoskeletal or cervical disorders, no history of surgery of the upper extremity and cervical region, no history of nerve injury or neurological impairment, no carpal or radial tunnel syndrome, and no rheumatoid disease or arthritis of the forearm and wrist during the 6 month preceding the study.

### Ethics Statement

All participants clearly understood the purpose of the study and signed consent forms. The study was approved by the Institutional Review Board (No. B-ER-101-003) of National Cheng Kung University Hospital.

### Instruments

**Fig 1:**
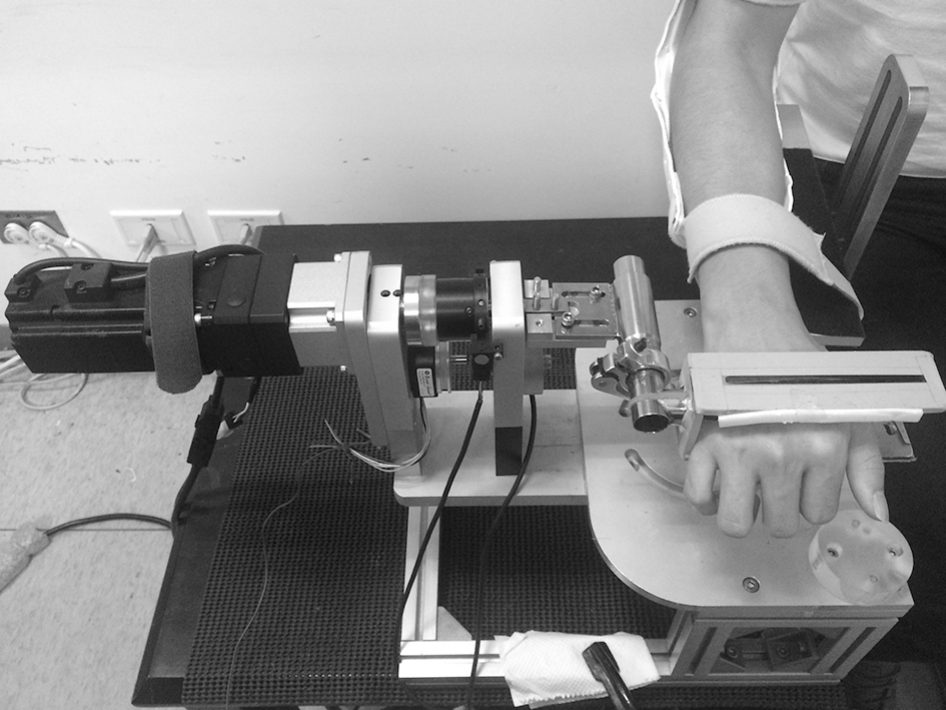
A custom-made bi-planar isokinetic dynamometer.

The BID has the functions of guiding the hand movement, detecting the wrist joint moment and controlling the movement velocity and wrist joint angle. The BID has two motion axes, including flexion/extension and ulnar/radial deviation of the wrist joint (Fig 1). The BID has high correlation (*r* = 0.99) in the static and dynamic calibration, and high consistency and reliability in measuring maximum voluntary isometric contraction (MVIC) at the neutral position (NP), radial deviation (RD), and ulnar deviation (UD) among three trials within a day and tests between different days (ICC>0.80). The muscle activity was recorded by a surface electromyography (EMG) system (MA300, Motion Lab Systems, Baton Rouge, LA USA). Bipolar stainless-steel disk surface electrodes (12 mm disks; MA-411, Motion Lab Systems, Baton Rouge, LA) were used to measure activity from the muscle bellies of the extensor digitorum communis (EDC) and flexor digitorum superficialis (FDS) [11]. The inter-electrode distance was 17 mm with a 13 × 3 mm reference electrode bar between the sensors. A ground electrode was placed at the head of the ulna on the wrist.

### Experimental procedure

Each subject first generated three MVIC trails of wrist extensors and flexors under NP, 20° UD, and 20° RD. After performing the MVIC test, the submaximal isokinetic contractions were conducted by the subjects so that they could be familiar with the formal isokinetic experiment. The maximal isokinetic concentric contractions of the wrist extensors and eccentric contractions of the wrist flexors were performed under NP, 20° UD, and 20° RD from 50° of wrist flexion to 40° of extension with the movement velocities of at 30°s^-1^, 90°s^-1^, and 180°s^-1^ during wrist extension and flexion. The maximal isokinetic eccentric contractions of the wrist extensors and concentric contractions of the wrist flexors were performed at three postures from 40° of wrist extension to 50° of flexion, with three movement velocities during wrist extension and flexion. The isokinetic concentric contraction was defined as the direction of exerted force, which is the same as the direction of dynamometer movement. The isokinetic eccentric contraction was defined as the direction of exerted force, which is the opposite as the direction of dynamometer movement. Two axes of rotation in the BID were aligned with the flexion-extension axis determined by the linkage between ulnar and radial styloid processes, and the abduction-adduction axis determined by the alignment between the third metacarpal bone and midline of the forearm.

### Data analysis

Peak torque (PT) was obtained from isokinetic contractions of wrist extensors/flexors and then normalized to MVIC, which represents the wrist joint moment. The ratio of normalized PT between wrist extensors and flexors was defined as the normalized PT of wrist extensors divided by the normalized PT of wrist flexors during isokinetic contractions at wrist extension and flexion, which represents the muscle balance of the wrist joint. The root mean square (RMS) of the EMG signal was calculated over the period in which the velocity was constant. The average RMS of EDC and FDS were normalized by the average RMS of EDC and FDS in MVIC at all movement velocities. The ratio of normalized RMS between EDC and FDS was defined as the normalized RMS of EDC divided by the normalized RMS of FDS during isokinetic contractions of wrist extensors, which represents the muscle co-activation.

### Statistics

Two-way ANOVA with repeated measures was used to compare the effects of movement velocity (30°s^-1^, 90°s^-1^, and 180°s^-1^) and wrist deviation (NP, UD, RD) on the normalized PT of wrist extensors, ratio of normalized PT between extensors and flexors, normalized RMS of EDC, and ratio of RMS between EDC and FDS during the isokinetic testing. There were two within-subject factors (movement velocity and wrist deviation). When significant overall main effects without interaction effects between wrist deviation and angular velocity were found, the post hoc test with an LSD was used to identify significant differences among the variables of movement velocity and those of wrist deviations. The level of significance was set at *P*<0.05. If interaction effects between wrist deviation and angular velocity were detected, a simple main effect of movement velocity and wrist deviation (one-way ANOVA with repeated measures) was employed separately with an LSD post hoc test. The level of significance with adjustment was set at *P*<0.016. The SPSS 17.0 statistical software (SPSS Inc., Chicago, IL, USA) was used for statistical analysis.

## Results

### Normalized PT of wrist extensors

**Fig 2:**
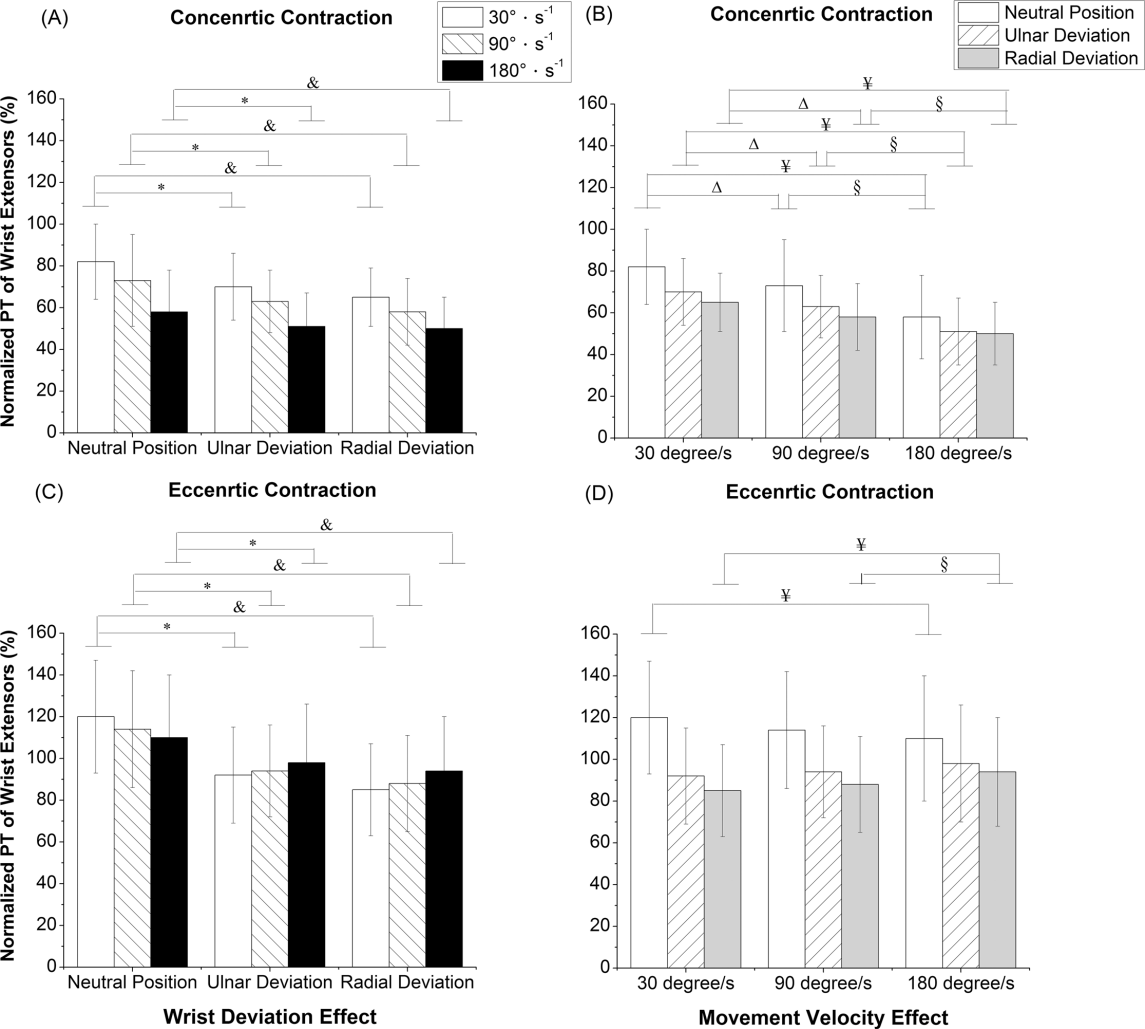
Normalized PT of wrist extensors. (A) concentric contraction with wrist deviation effect, (B) concentric contraction with movement velocity effect, (C) eccentric contraction with wrist deviation effect, and (D) eccentric contraction with movement velocity effect, PT: peak torque. ^&^Significant differences in wrist deviation between the neutral position and radial deviation with *P < 0*.*05*; *Significant differences in wrist deviation between the neutral position and ulnar deviation with *P < 0*.*05*; ^Δ^Significant differences in movement velocity between 30°s^-1^ and 90°s^-1^ with *P < 0*.*05*; ^¥^Significant differences in movement velocity between 30°s^-1^ and 180°s^-1^ with *P < 0*.*05*; ^§^Significant differences in movement velocity between 90°s^-1^ and 180°s^-1^ with *P < 0*.*05*.

The interaction effects between wrist deviation and movement velocity were statistically significant in the normalized PT of wrist extensors during concentric (*P* = 0.028) and eccentric (*P* < 0.001) contractions. With a simple main effect of wrist deviation, the normalized PT of NP was significantly larger than those under RD and UD during the concentric contraction at 30°s^-1^ (*P* < 0.001, *P* < 0.001), 90°s^-1^ (*P* < 0.001, *P* = 0.001), and 180°s^-1^ (*P* = 0.011, *P* = 0.007) (Fig 2(A)). With regard to the simple main effect of movement velocity, the normalized PT of extensors at 30°s^-1^ were significantly greater than those at 90°s^-1^ and 180°s^-1^ under NP (*P* < 0.001, *P* < 0.001), RD (*P* < 0.001, *P* < 0.001), and UD (*P* < 0.001, *P* < 0.001) during the concentric contraction (Fig 2(B)). During the eccentric contraction, the normalized PT of NP was significantly larger than those under RD and UD at 30°s^-1^ (*P* < 0.001, *P* < 0.001), 90°s^-1^ (*P* < 0.001, *P* < 0.001), and 180°s^-1^ (*P* < 0.001, *P* < 0.001) (Fig 2(C)). The normalized PT of extensors at 180°s^-1^ were significantly larger than at 30°s^-1^ (*P* = 0.009) and 90°s^-1^ (*P* = 0.014) under RD during the eccentric contraction (Fig 2(D)). Otherwise, the normalized PT of extensors at 180°s^-1^ were significantly smaller than at 30°s^-1^ (*P* = 0.006) under NP during the eccentric contraction (Fig 2(D)).

### Ratio of normalized PT between wrist extensors and flexors

**Fig 3:**
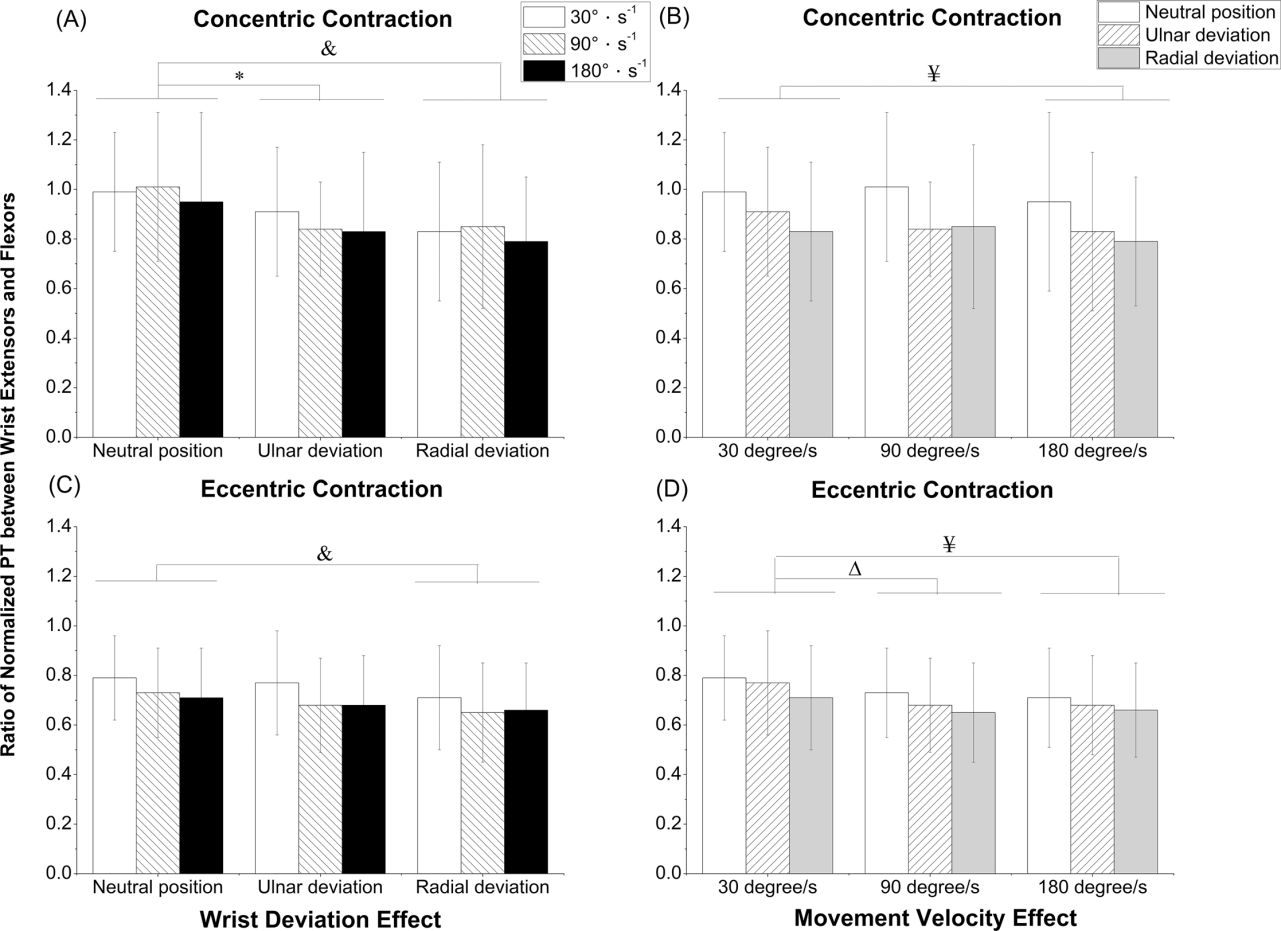
Ratio of normalized PT between wrist extensors and flexors. (A) concentric contraction with wrist deviation effect, (B) concentric contraction with movement velocity effect, (C) eccentric contraction with wrist deviation effect, and (D) eccentric contraction with movement velocity effect, PT: peak torque. ^&^Significant differences in wrist deviation between the neutral position and radial deviation with *P < 0*.*05*; *Significant differences in wrist deviation between the neutral position and ulnar deviation with *P < 0*.*05*; ^Δ^Significant differences in movement velocity between 30°s^-1^ and 90°s^-1^ with *P < 0*.*05*; ^¥^Significant differences in movement velocity between 30°s^-1^ and 180°s^-1^ with *P < 0*.*05*.

There were no significant interaction effects between wrist deviation and movement velocity in the ratio of normalized PT between extensors and flexors during both contraction types. The overall main effect of wrist deviation (*P* < 0.001) and movement velocity (*P* = 0.039) showed statistical significance in the ratio of normalized PT between wrist extensors and flexors during the concentric contraction. Moreover, the overall main effect of wrist deviation (*P* = 0.010) and movement velocity (*P* < 0.001) showed statistical significance in the ratio of normalized PT between wrist extensors and flexors during the eccentric contraction. The NP had significantly greater values in comparison with RD (*P* = 0.001) and UD (*P* = 0.009) during the concentric contraction (Fig 3(A)); moreover, the NP also had a significantly larger values than RD (*P* = 0.005) during the eccentric contraction (Fig 3(C)). Significant differences in the ratios between 30°s^-1^ and 180°s^-1^ were found during the concentric (*P* = 0.025, Fig 3(B)) and eccentric (*P* < 0.001) contractions (Fig 3(D)).

### Normalized RMS of EDC

**Fig 4:**
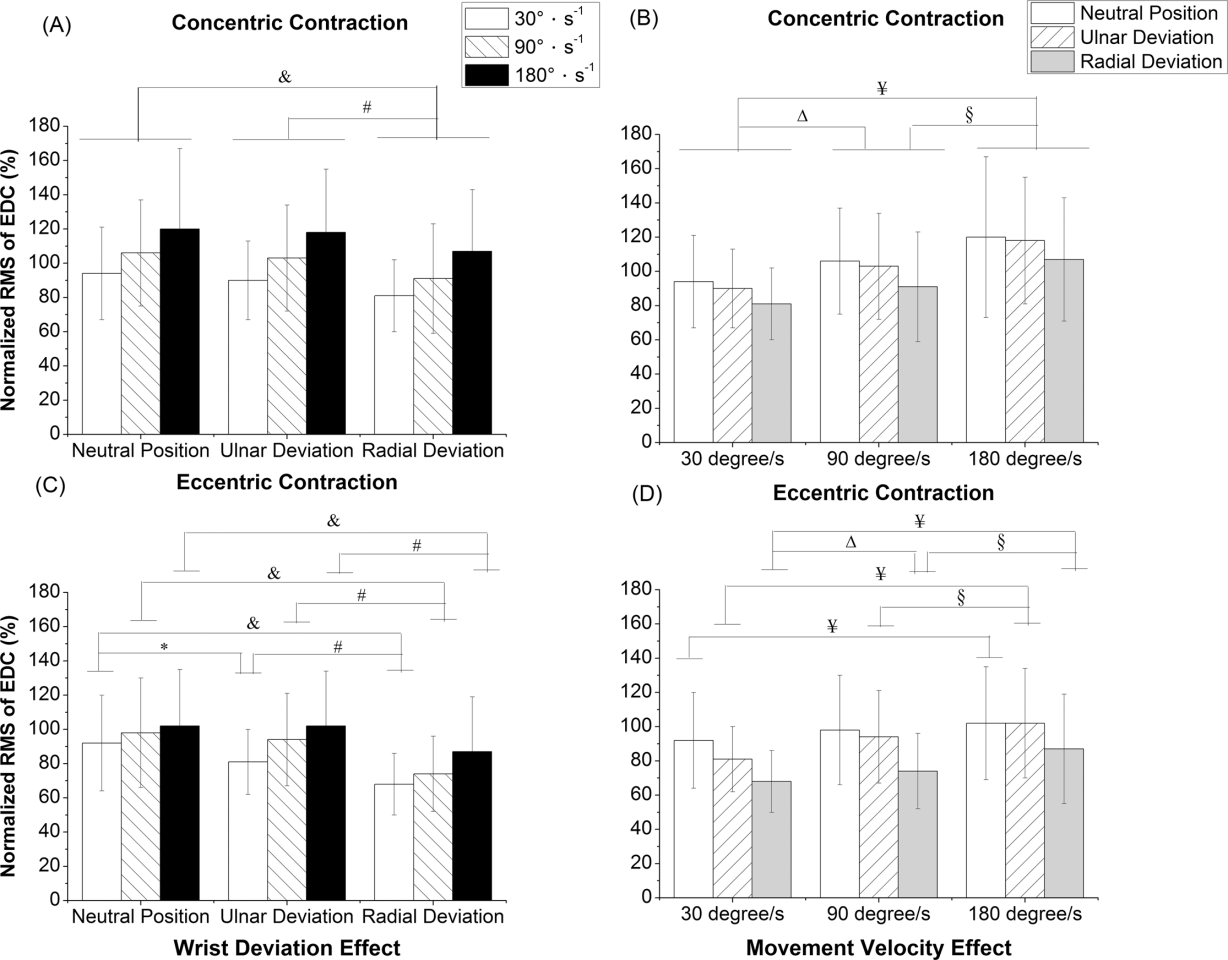
Normalized RMS of EDC. (A) concentric contraction with wrist deviation effect, (B) concentric contraction with movement velocity effect, (C) eccentric contraction with wrist deviation effect, and (D) eccentric contraction with movement velocity effect, RMS: root mean square. ^&^Significant differences in wrist deviation between the neutral position and radial deviation with *P < 0*.*05*; *Significant differences in wrist deviation between the neutral position and ulnar deviation with *P < 0*.*05*; ^#^ Significant differences in wrist deviation between the radial deviation and ulnar deviation with *P < 0*.*05*; ^Δ^Significant differences in movement velocity between 30°s^-1^ and 90°s^-1^ with *P < 0*.*05*; ^¥^Significant differences in movement velocity between 30°s^-1^ and 180°s^-1^ with *P < 0*.*05*; ^§^Significant differences in movement velocity between 90°s^-1^ and 180°s^-1^ with *P < 0*.*05*.

There were no significant interaction effects between wrist deviation and movement velocity in the normalized RMS of EDC during the concentric contraction. The overall main effect of wrist deviation (*P* < 0.001) and movement velocity (*P* < 0.001) showed statistical significance in the normalized RMS of EDC during the concentric contraction. The normalized RMS of EDC under RD was significantly smaller than those under NP (*P* = 0.001) and UD (*P* < 0.001) during the concentric contraction (Fig 4(A)). The normalized RMS of EDC at the 180°s^-1^ was larger than at 90°s^-1^ (*P* < 0.001) and 30°s^-1^ (*P* < 0.001) during the concentric contraction (Fig 4(B)). Significant interaction effects (*P* = 0.006) between wrist deviation and movement velocity were found in the normalized RMS of EDC during the eccentric contraction. The normalized RMS under NP was larger than RD at 30°s^-1^ (*P* < 0.001), 90°s^-1^ (*P* < 0.001), and 180°s^-1^ (*P* = 0.004) during the eccentric contraction (Fig 4(C)). In light of a simple main effect of movement velocity, the normalized RMS of EDC at 180°s^-1^ had a greater value than 30°s^-1^ under NP (*P* = 0.012), RD (*P* < 0.001), and UD (*P* < 0.001) during the eccentric contraction (Fig 4(D)).

### Ratio of normalized RMS between EDC and FDS

**Fig 5:**
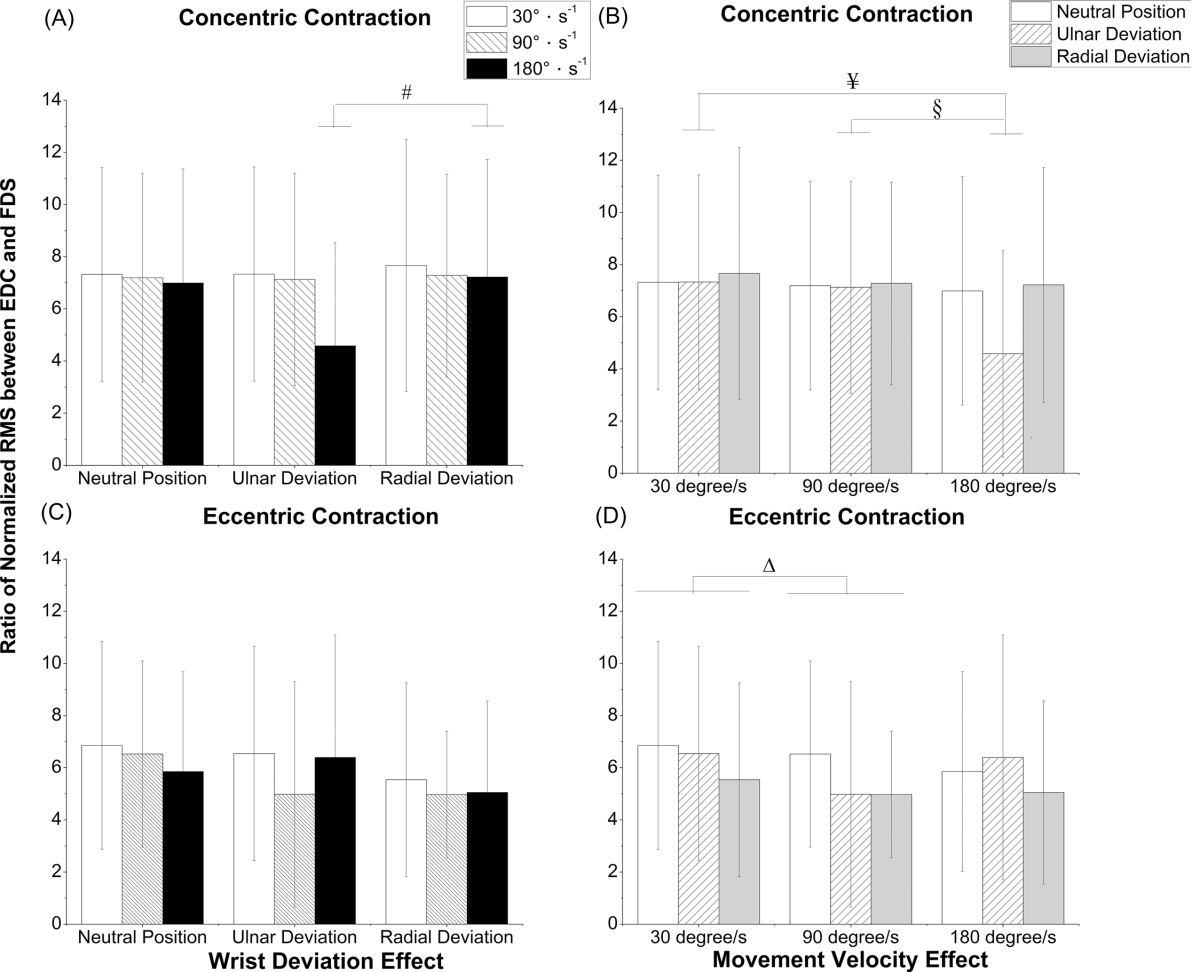
Ratio of normalized RMS between EDC and FDS. (A) concentric contraction with wrist deviation effect, (B) concentric contraction with movement velocity effect, (C) eccentric contraction with wrist deviation effect, and (D) eccentric contraction with movement velocity effect, RMS: root mean square. ^#^Significant differences in wrist deviation between the radial deviation and ulnar deviation with *P < 0*.*05*; ^Δ^Significant differences in movement velocity between 30°s^-1^ and 90°s^-1^ with *P < 0*.*05*; ^¥^Significant differences in movement velocity between 30°s^-1^ and 180°s^-1^ with *P < 0*.*05*; ^§^Significant differences in movement velocity between 90°s^-1^ and 180°s^-1^ with *P < 0*.*05*.

Significant interaction effects (*P* = 0.006) between wrist deviation and movement velocity were revealed in the ratio of normalized RMS between EDC and FDS in the concentric contraction. The ratio of normalized RMS between EDC and FDS had a significantly greater value in the wrist deviation under RD than UD (*P* = 0.003) at 180°s^-1^ during the concentric contraction (Fig 5(A)). The ratio was significantly greater at 30°s^-1^ than that at 180°s^-1^ (*P* = 0.002), and greater at 90°s^-1^ than that at 180°s^-1^ (*P* = 0.002) under UD during the concentric contraction (Fig 5(B)). No significant interaction effects between wrist deviation and movement velocity were revealed in the ratio of normalized RMS between EDC and FDS during the eccentric contraction. The overall main effect of movement velocity (*P* = 0.026) showed statistical significance in the ratio of normalized RMS between EDC and FDS during the eccentric contraction; nevertheless, there was no statistical significance in the overall main effect of wrist deviation. During the eccentric contraction, the ratio of normalized RMS between EDC and FDS at 30°s^-1^ had a significantly greater value than at 90°s^-1^ (*P* = 0.026, Fig 5(D)).

## Discussion

### Velocity effect: Muscle strength

This study showed that the normalized PT during isokinetic concentric and eccentric contractions was influenced by the movement velocity. Based on the force-velocity relationship in skeletal muscles, the muscle strength decreases with increased angular velocity in the concentric contraction; however, the muscle strength increases as the velocity rises in the eccentric contraction [12, 13]. The force-velocity relationship is supported by many factors that have a muscle physiologic basis, such as the pattern of motor unit recruitment [12] and condition of cross-bridge overlap [13]. Ellenbecker et al. reported a higher peak torque of wrist extensors at a lower velocity in comparison to a higher velocity when performing isokinetic concentric contraction in elite tennis players [14]. The findings of our study showed similar results, which demonstrate the normalized PT of extensors decreased when increasing the movement velocity in the concentric contraction at all postures. Furthermore, the normalized PT of extensors during the eccentric contraction was significantly increased in proportion to the increase in movement velocity at the UD and RD, but not for the NP. The isokinetic performance of eccentric contraction at NP was not related to the force-velocity relationship, due not only to intrinsic factors of the muscle and joint, but also neuromuscular factors. The maximum torque production of the wrist joint was influenced by intrinsic factors such as the moment arm, muscle force, sacromere length, and tendon strain [15]; moreover, the patterns of motor unit recruitment [12] and fiber type distribution can also alter the torque production. In addition, previous studies proposed that the inhibition of the neural system is one of the factors that can result in reduced torque production during eccentric contraction in order to prevent muscle injury [16–18].

The ratio of muscle strength between the agonist and antagonist is affected by physiological properties of individuals, angular velocity [19] and joint angle [20, 21] during isokinetic testing. Previous research showed that the ratio of muscle strength between hamstring and quadriceps in the concentric contraction increases when raising the joint movement velocity, and that it had a constant value when the velocity increased in the eccentric contraction [20]. Ellenbecker et al. reported that tennis players had smaller ratio of strength between wrist extensors and flexors on the non-dominant side at a fast velocity [14]. This study found similar results to the studies which demonstrated a decrease in the strength ratio between wrist extensors and flexors at a fast velocity in both contraction types. The muscle imbalance between agonist and antagonist might change the joint moment, joint loading and muscle activity, which may then result in muscular injuries. Muscle imbalance of the isokinetic contraction profile has been rarely investigated in the wrist joint, although the muscle imbalance of isometric contraction in the wrist joint has been often studied for patients with tennis elbow [5] or elite tennis players [14]. The specific patterns with regard to the balance of muscle strength between the agonist and antagonist could provide information regarding injury prevention or strengthening guidelines for the wrist joint. According to our study, when there was an increase in the movement velocity or performance of the wrist extension combined with deviation, the ratio of normalized PT between the wrist extensors and flexors was decreased. This indicates that the strength of the wrist extensors was lower than that of the flexors during rapid movements and the coupling posture. When performing a quick movement with the wrist coupling posture during work or exercise, the results of this study suggest there is a need to train the muscle strength of the wrist extensors at high velocity with coupling movement via the bi-planer isokinetic dynamometer. Furthermore, patients with lateral epicondylitis could strengthen their wrist extensors to achieve a ratio of normalized PT between extensors and flexors of 1.0 at an angular velocity of 90°s^-1^ during the concentric contraction, or a ratio of 0.7 at an angular velocity of 90°s^-1^ during the eccentric contraction at neutral position, based on the findings of this study.

### Velocity effect: Muscle activity

According to the findings of this study, the muscle activity of EDC is velocity-dependent, with the results showing that the normalized RMS of EDC increased with rising velocity in both contraction types of the wrist movement. Previous research indicated that the force-velocity relationship of the skeletal muscle might not be able to comprehensively explain the muscle activity under different angular velocities and contraction types [22]. It also noted that this phenomenon might be influenced by other intrinsic factors, such as muscle contractile properties. The findings of the current study suggested that EDC generated higher activation as the velocity increased to complete dynamic movement, regardless of the contraction type. The pattern of co-activation between the agonist and antagonist is related to the joint stability, and associated with the velocity of movement. A previous study showed a higher co-activation between antagonist and agonist as the velocity rises in the knee joint [23]. The current study indicated that the co-activation of the antagonist demonstrated relatively higher muscle activity, due to the fast velocity needed to maintain the stability of the wrist joint in the concentric contraction. The possible reason for this is the ability of neuromuscular control, which tries to decrease the kinematic variability and maintain the stability of the wrist joint [24].

### Wrist deviation effect: Muscle strength

Many workers and athletes require a variety of coupling movements in the wrist joint during performance. Workers who have lateral epicondylitis often engage in excessive physical exertions combined with awkward wrist postures for more than two hours per day [2]. Tennis elbow often occurs due to the wrist posture in ulnar deviation and wrist flexion with a long duration of eccentric contraction during the backhand stroke [10]. The use of an awkward posture and unskilled technique with specific contraction characteristics of the muscles could thus induce tennis elbow. The findings of this study indicate that wrist postures significantly changed the torque of wrist extensors and ratio of torque between extensors and flexors in both contraction types at all movement velocities (NP>UD>RD). Alternating muscle length in the RD and UD of the wrist joint might be one of factors to produce the relatively lower strength of the wrist extensors due to anatomical features. Therefore, when the wrist deviates from the NP to RD/UD, the length of the wrist extensors changes and results in an unsuitable muscle length to generate force. A previous study indicated that the extensor carpi radialis brevis showed a more optimal muscle length in pronation than the neutral position of the forearm [25]. Accordingly, such awkward postures could cause a vicious cycle of injuries in the muscular system, mainly due to the mechanical disadvantage of inappropriate muscle length and moment arm. Wrist deviation has been reported to produce decreased grip and pinch forces [26], thus influencing functional performance in daily activities. Furthermore, deviation of the wrist joint with a high level of repetitive movement could result in the poorer prognosis of elbow tendonitis [27].

### Wrist deviation effect: Muscle activity

The muscle activity of EDC during wrist extension was lower at RD than UD and NP in both contraction types at all movement velocities. Moreover, the normalized PT of wrist extensors showed a similar pattern with muscle activity of the wrist extensors during isokinetic contraction at different postures. The results of this study indicate that the level of muscle activity in EDC is related to the level of force generated, which agrees with the findings in previous works [28, 29]. The decrease in muscle activity of the wrist extensors may be caused by the alternation of the wrist angle in the UD and RD. A previous study indicated that wrist posture resulted in forearm muscle shortening, which would alter muscle activity during the handgrip movement [30]. Otherwise, the wrist neutral position was at 5–7° ulnar deviation and 7–9° extension, which significantly decreased the muscle activity of the forearm muscles compared with extreme wrist deviations [31]. The extreme deviation of the wrist joint may cause overstretch in wrist muscles and alter the activity of wrist muscles during dynamic performances, which may induce musculoskeletal injury of the elbow joint. The ratio of muscle activity between the agonist and antagonist is relatively high in the RD in comparison with the UD and NP during concentric contraction. However, during eccentric contraction the RD showed a lower ratio of muscle activity than UD and NP, except for the 90°s^-1^ movement velocity. This suggests that co-activation of the antagonist demonstrates relatively low activity in the RD, which means wrist flexors could not generate a higher level of muscle activity to maintain the stability of the wrist joint in the concentric contraction. Moreover, the antagonist had relatively higher activity at the RD to keep the wrist stable during eccentric contraction. Patients with tennis elbow suffer from an imbalance of the forearm muscles which reduces muscle activity of the extensor carpi radialis [6]. Extreme wrist deviation combined with extension would reduce the muscle activity of the EDC and alter the balance between the EDC and FDS, which may result in injury of the upper extremities in response to maximum eccentric contraction force.

With regard to the clinical relevance of this work, the findings suggest that a wrist deviation combined with extension may produce a mechanical disadvantage with regard to performing the required task with exerted force, due to the lower strength and muscle activity at the RD of the wrist joint during the isokinetic contraction. We thus suggest that strengthening of the wrist extensors in dynamic movement with different wrist postures should be a goal of strength training in manual workers or athletes to prevent injury in the upper extremities. Nevertheless, the muscle balance between the agonist and antagonist is another important factor in controlling the dynamic performance, and thus all the wrist extensors and flexors need to be trained simultaneously and dynamically at different wrist coupling postures for the rehabilitation of upper extremity disorders. The establishment of normative data achieved in this work may have important implications with regard to rehabilitation treatment, strength training and functional performance in forearm muscles during coupling movements for healthy subjects and patients with muscular-skeleton system disorder in the upper extremities.

One factor which limits the interpretation of the results is the use of surface EMG to measure the muscle activity only in the EDC and FDS. The reason for this was to prevent the cross-talk effect of the EMG signals among the small muscle groups of the forearm. In addition, when investigating the effects of wrist deviation and movement velocity on muscle strength and activity, the effect of gender should also be considered in future work, as this is another factor that influences the pattern of muscle strength and activity during wrist extension, along with the deviation in the isokinetic contraction.

## Conclusion

The wrist deviation combined with extension and movement velocity of the wrist joint should be considered as the influential factors which might alter the wrist strength and muscle activity during the dynamic performance, and then may result in injury of the elbow joint further. Manual workers or athletes who generate exerted force should thus avoid extreme coupling postures of the wrist joint with rapid contraction movements due to the reduced value of the RMS of EDC and torque of the wrist extensors in deviation when compared to the neutral position, and decreased ratio of the RMS between EDC and FDS and ratio of strength between the wrist extensors and flexors at fast velocity. Clinicians and athletic trainers could use the BID to evaluate the dynamic performances of wrist extensors and flexors at different coupling postures and movement velocities for athletes with or without musculoskeletal injury of the upper extremities, enabling them to improve their functional performance and decrease the risk of serious injury.
